# Neutrophil–Creatinine Index: A New Prognostic Factor for Severity of Acute Pancreatitis

**DOI:** 10.3390/medicina60040607

**Published:** 2024-04-06

**Authors:** Abdurrahman Sahin

**Affiliations:** Gastroenterology Department, Faculty of Medicine, Tokat Gaziosmanpasa University, 60030 Tokat, Turkey; arahmansmd@yahoo.com; Tel.: +90–506-511–6191

**Keywords:** acute pancreatitis, neutrophil–creatinine index, neutrophil to lymphocyte ratio, prognosis, severity

## Abstract

*Background and Objectives*: Determining the severity of acute pancreatitis (AP) is the main goal in the early stage of AP. The aim of this study was to compare laboratory parameters and indices, including the neutrophil to lymphocyte ratio (NLR) and the neutrophil–creatinine index (NCI), at admission in order to predict the severity of AP. *Materials and Methods*: Data from 421 patients who were admitted with a diagnosis of AP were collected retrospectively. Disease severity was assessed using the Bedside Index of Severity in Acute Pancreatitis (BISAP) and the revised Atlanta classification (RAC). BISAP was graded as mild and severe, and RAC was graded as mild (MAP), moderately severe (MSAP), and severe (SAP). The laboratory parameters and indices, including the NLR and NCI, were compared. *Results*: Of the patients, 70 (16.6%) had severe AP according to BISAP; the AP subgroups according to the RAC were as follows: MAP (*n* = 213), MSAP (*n* = 158), and SAP (*n* = 50). The NCI had the highest area under the receiver operator characteristic (AUROC) curve value (0.862), demonstrating severe disease according to BISAP, with a sensitivity of 78.6% and a specificity of 79.8%. Age (OR:1.046), white blood cell count (WBC) (OR:1.141), hematocrit (OR:1.081), blood urea nitrogen (BUN) (OR:1.040), and NCI (OR:1.076) were independently associated with severe disease, according to the multivariate analysis results, and were determined as components of the newly developed nomogram. The AUROC of the nomogram (0.891) was superior to the AUROCs of all the components of the nomogram except the NCI. Moreover, the NCI was the only parameter to distinguish MSAP from MAP (OR:1.119, 95% CI: 1.015–1.235, *p* = 0.023) and SAP from MSAP (OR:1.095, 95% CI: 1.031–1.162, *p* = 0.003). *Conclusions*: The present study enabled the identification of the neutrophil–creatinine index as a new prognostic tool for the assessment of AP severity at hospital admission.

## 1. Introduction

Acute pancreatitis (AP) is an inflammatory disorder of the pancreatic tissue that is usually accompanied by severe abdominal pain, nausea, and vomiting. Although the clinical course of AP is mild and self-limiting in most patients, up to 20% of these patients develop severe AP, which includes manifestations of systemic inflammatory response syndrome (SIRS) and multiple organ failure (MOF). The overall mortality of AP is 5%, and this rate may be as high as 25% in those with severe AP [1]. The best predictor of poor outcomes in AP is the development of persistent MOF and pancreatic necrosis. Therefore, it is important to predict the course and severity of the disease at an early stage.

The most widely used classification systems for determining the severity and course of AP are the revised Atlanta classification and the Bedside Index of Severity in Acute Pancreatitis (BISAP). According to the revised Atlanta classification, the severity of AP is graded as mild acute pancreatitis (MAP), moderately severe acute pancreatitis (MSAP), or severe acute pancreatitis (SAP) [2]. In addition to the revised Atlanta classification and BISAP, several other prognostic scoring systems and classifications have been developed to predict the severity of AP [3]. Ranson’s criteria, the Acute Physiology and Chronic Health Evaluation (APACHE) II score, the Modified Glasgow Prognostic Score, and the Balthazar index are other commonly used prognostic systems. Most of these scoring systems include multiple determinants or parameters that must be noted 24 to 48 h after hospitalization, and the estimation of the severity of AP is delayed until 48 h after hospitalization [4]. Thus, these scoring systems are of limited use at admission [5].

On the other hand, in view of the complexity of prognostic scoring systems, several studies have been conducted on the role of simple laboratory parameters and indices in predicting the disease severity of AP and mortality. The most widely studied laboratory parameters and indices are the white blood cell count (WBC), neutrophil count, neutrophil to lymphocyte ratio (NLR), platelet to lymphocyte ratio (PLR), hematocrit, blood urea nitrogen (BUN), creatinine, calcium, C-reactive protein (CRP), and procalcitonin [5]. These laboratory parameters have also been used as part of several prognostic scoring systems. Nevertheless, none of the laboratory parameters or prognostic scoring systems can predict the severity of AP or MOF with sufficient accuracy [6,7].

The first 48 h after the onset of symptoms is very important; during this period, it is necessary to identify the patients at risk for the development of complications or even death. This period is a crucial time in which to determine the aggressiveness of the required treatment, which includes fluid resuscitation, pain control, and nutritional support. Therefore, it is important to decide upon admission whether the patients require close monitoring or transfer to an intensive care unit (ICU). Moreover, it is not easy to predict the course and severity of acute pancreatitis at the first assessment of hospital admission with laboratory parameters or scoring systems other than BISAP. At the time of admission, most laboratory parameters are insufficient to differentiate mild disease from severe disease, and changes in laboratory parameters over time, including inflammatory markers and parameters related to organ/system dysfunction, provide important clues regarding the course of the disease. On the other hand, we need simple, reliable, widely used parameters at admission for predicting the course of the disease.

We found a new index named the neutrophil–creatinine index, which was established based on the levels of neutrophil count and creatinine, to predict the severity of AP at admission. Neutrophil infiltration and activation is one of the early events in acute pancreatitis and results in higher neutrophil count values at admission. Similarly, higher creatinine values at admission indicate renal hypoperfusion, which is associated with third-space leakage resulting from a systemic inflammatory state. We assumed that the NCI may serve as an efficient test to represent the combination of inflammatory response and organ dysfunction. Therefore, in the current study, we aimed to assess the clinical usefulness of the NCI as an early indicator of disease severity at hospital admission.

## 2. Materials and Methods

### 2.1. Study Design

This retrospective study was conducted in all consecutive adult patients who had been hospitalized with a diagnosis of AP at the Gastroenterology Clinic of Tokat Gaziosmanpasa University Hospital in Turkey between January 2018 and December 2021. Patients with recurrent pancreatitis were enrolled only at their first admission. The AP diagnosis was made according to the presence of at least two of the following three criteria: abdominal pain consistent with AP, serum amylase or lipase values at least three times greater than the upper limit of the normal value, and the characteristic findings of AP in radiological imaging studies. The exclusion criteria among the patients diagnosed with AP were pregnancy, end-stage renal disease, hematological disorders, pancreatic carcinoma or cholangiocarcinoma, and incomplete records.

The severity of AP was primarily determined using the BISAP score and the revised Atlanta classification. The BISAP score consists of five parameters, including an age >60 years, elevated BUN (>25 mg/dL), existence of pleural effusion, and development of systemic inflammatory response [8]. A BISAP score of 3 or more was interpreted as severe AP. The BISAP score was assessed in the first 24 h following admission. Furthermore, the patients were divided into three subgroups according to the revised Atlanta classification. The revised Atlanta classification is graded as follows: mild AP (MAP) with no organ failure and no local or systemic complications; moderately severe AP (MSAP) with transient organ failure that is resolved within 48 h following admission or local complications; and SAP with persistent organ failure, which is defined as organ failure that continues for longer than 48 h [2]. Moreover, Ranson’s criteria scores were also noted from hospital records.

The patient data, including age, gender, and AP etiology, and the laboratory data at admission were extracted from the hospital database. The recorded laboratory data comprised hemogram parameters, including WBC, neutrophil count, lymphocyte count, hemoglobin, hematocrit, platelet count, and routine biochemical tests, including glucose, aspartate amino transferase (AST), alanine amino transferase (ALT), bilirubin, calcium, BUN, creatinine, CRP, amylase, and lipase. The NLR was defined as the ratio of the neutrophil count to the lymphocyte count, and the PLR was defined as the ratio of the platelet count to the lymphocyte count. The neutrophil–creatinine index (NCI) was calculated as follows:

- NCI = neutrophil count (×103/μL) × creatinine (mg/dL)

### 2.2. Statistical Analysis

The results were analyzed using SPSS software 20.0 and MedCalc software 22.0. Normality was assessed by means of the Shapiro–Wilk test. For quantitative variables, data were presented as the mean ± standard deviation for normally distributed data and median ± standard error of the mean or median (interquartile range (IQR)) for non-normally distributed data. The groups were compared using the Student’s *t* test or the Mann–Whitney U test (2 categories) or ANOVA or the Kruskal–Wallis test (>2 categories). In the case of qualitative variables, the associations were verified using the Chi-square test or Fisher’s exact test. The area under the receiver operator characteristic (AUROC) curve was used to identify optimal cut-off values for NCI, NLR, and other laboratory parameters to recognize maximum sensitivity, specificity, positive predictive value (PPV), negative predictive value (NPV), and accuracy for AP severity. The DeLong test was used to compare the AUROCs. Multivariate logistic regression analysis was performed to evaluate the independent predictive value for AP severity among the variables that showed significant differences in univariate analysis. A nomogram was created according to the regression analysis results using a Python-based online program (https://visdata.bjmu.edu.cn/nomogram) (accessed on 18 January 2024) to assess the severity of AP. The discrimination of the model was assessed using the AUROC curve. A *p* value of less than 0.05 indicated statistical significance.

## 3. Results

### 3.1. Baseline Characteristics of the Study Population

A total of 421 patients with AP were included in this study. According to the etiological classification, 310 patients (73.6%) were in the biliary group and 51 (11.9%) were in the non-biliary group; no etiological factors were found in 60 patients (14.5%) and classified in the idiopathic group. The overall mean age of the patients was 62 ± 18 years, and 251 patients (60%) were female. Seventy patients (16.6%) had severe AP according to BISAP. Moreover, 213 patients (51%) were classified as being in the MAP group, 158 (37%) as being in the MSAP group, and 50 (12%) as being in the SAP group, according to the revised Atlanta classification. Death was recorded in 18 patients (4.3%). The baseline characteristics of the patients are summarized in Table 1.

### 3.2. Comparison of Laboratory Parameters Related to AP Severity According to BISAP

#### 3.2.1. Laboratory Parameters to Distinguish Mild AP from Severe AP

When the patients were compared in terms of severity according to BISAP, the WBC, neutrophil count, hemoglobin, hematocrit, BUN, creatinine, CRP, NLR, and NCI levels were higher among the severe cases. Comparisons of these laboratory parameters are presented in Table 2.

#### 3.2.2. Prognostic Accuracy of NCI and Other Laboratory Parameters

Regarding the prediction of AP severity according to BISAP, we investigated WBC, BUN, hematocrit, CRP, NLR, and NCI using ROC analysis of the statistically significant laboratory parameters. The AUROCs for the laboratory parameters are shown in Table 3. For an NCI value of 11.27, the sensitivity, specificity, PPV, NPV, and accuracy were 78.6%, 79.8%, 43.7%, 94.9%, and 79.6%, respectively. The ability of NCI to distinguish severe AP from mild AP was superior to that of BUN (*p* = 0.016), WBC (*p* = 0.002), CRP, hematocrit, and NLR (for all, *p* < 0.001). When compared, the AUROCs of the combinations of NCI with WBC, BUN, hematocrit, CRP, and NLR (0.862, 0.877, 0.862, 0.862, and 0.862, respectively) did not differ from the AUROC of NCI (for all, *p* > 0.05).

Table 4 displays the results of the logistic regression analysis examining the factors associated with severe disease. The univariate analysis revealed that age (OR:1.012, 95% CI, 1.001–1.024, *p* = 0.003), female gender (OR:0.508, 95% CI, 0.303–0.852, *p* = 0.01), WBC (OR:1.232, 95% CI, 0.02–0.27, *p* = 0.023), hematocrit (OR:1.095, 95% CI, 1.043–1.149, *p* = 0.033), BUN (OR:1.058, 95% CI, 1.038–1.078, *p* < 0.001), CRP (OR:1.006, 95% CI, 1.003–1.010, *p* < 0.001), NLR (OR:1.178, 95% CI, 1.126–1.232, *p* < 0.001), and NCI (OR:1.041, 95% CI, 1.131–1.257, *p* < 0.001) were associated with severe AP. In the multivariate analysis, age (OR:1.046, 95% CI, 1.018–1.074, *p* = 0.001), WBC (OR:1.141, 95% CI, 1.026–1.269, *p* = 0.015), hematocrit (OR:1.081, 95% CI, 1.015–1.152, *p* = 0.015), BUN (OR:1.040, 95% CI, 1.014–1.066, *p* = 0.002), and NCI (OR:1.076, 95% CI, 1.014–1.142, *p* = 0.015) were independent risk factors for severe AP.

**Figure 1:**
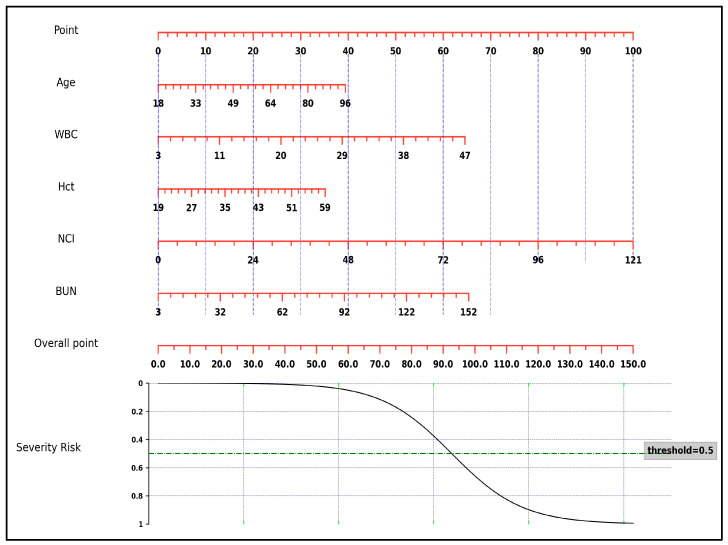
Nomogram estimating the likelihood of severity in patients with acute pancreatitis. BUN, blood urea nitrogen; Hct, hematocrit; NCI, neutrophil–creatinine index; WBC, white blood cell count.

**Figure 2:**
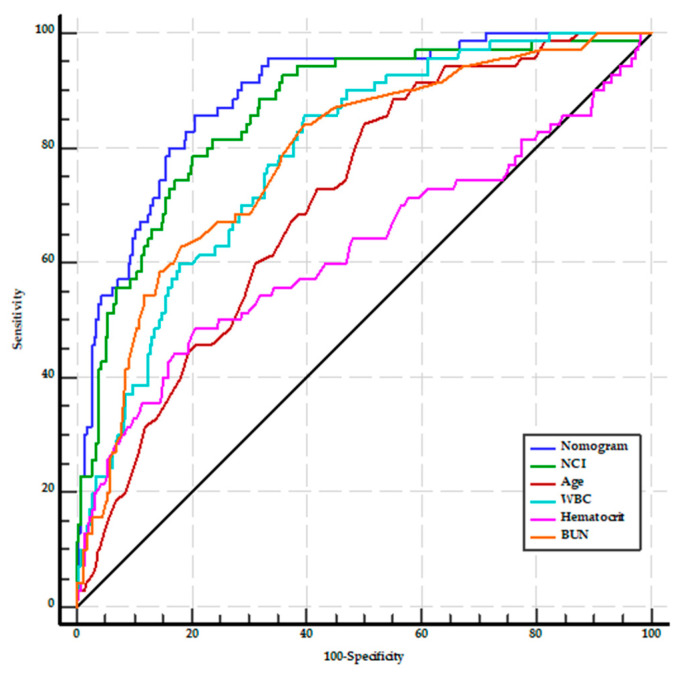
AUROCs for the prediction of severe acute pancreatitis at admission. BUN, blood urea nitrogen; NCI, neutrophil–creatinine index; WBC, white blood cell count.

A nomogram was constructed using the results of the multivariate analysis to estimate the likelihood of severity in patients with AP (Figure 1). The scores were constructed using five independent factors, and the estimated severity risk was calculated by summing the scores of each factor with the weight equal to the OR value. The final score ranged from a minimum of 0 points to a maximum of 150 points. The AUROC of the nomogram was 0.891 (95% CI: 0.858–0.919), with sensitivity, specificity, PPV, NPV, and accuracy of 85.7%, 79.5%, 45.5%, 96.5%, and 80.5%, respectively. It was higher than the AUROC values for age (0.709, 95% CI: 0.663–0.752), WBC, BUN, and hematocrit (for all, *p* < 0.001). On the other hand, the AUROC of the nomogram did not significantly differ from the AUROC of the NCI (*p* = 0.077) (Figure 2).

### 3.3. Comparison of Laboratory Parameters Related to AP Severity According to the Revised Atlanta Classification Subgroups

#### 3.3.1. Laboratory Parameters for Distinguishing AP Subgroups

The laboratory values of the subgroups that were defined according to the revised Atlanta classification are presented in Table 5. Among these, WBC, neutrophil count, creatinine, and NCI were the parameters that differentiated the three subgroups. While hematocrit and NLR distinguished the cases in the MAP subgroup from those in the MSAP and SAP subgroups, BUN and CRP distinguished the SAP subgroup from the other two subgroups.

#### 3.3.2. Prognostic Accuracy of NCI and Other Laboratory Parameters to Distinguish MSAP from MAP

**Figure 3:**
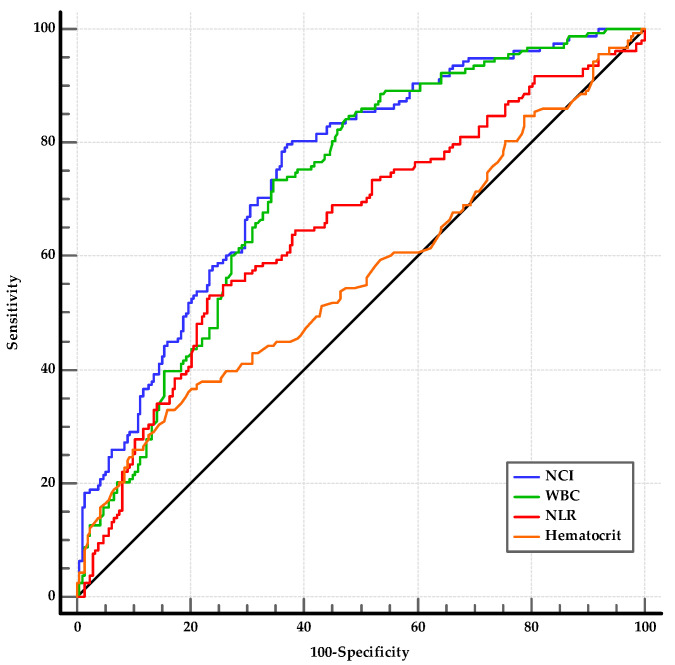
AUROCs of NCI, NLR, WBC, and hematocrit for distinguishing MSAP from MAP. NCI, neutrophil–creatinine index; NLR, neutrophil to lymphocyte ratio; WBC, white blood cell count.

Regarding the studied laboratory parameters, the WBC, neutrophil, hemoglobin, hematocrit, creatinine, NLR, and NCI levels were significantly higher in the MSAP group than in the MAP group (for all, *p* < 0.05) (Table 5). Among the statistically significant laboratory parameters, WBC, hematocrit, NLR, and NCI were investigated using ROC analysis. The optimal cut-offs for predicting the MSAP group were as follows: WBC > 10,840/mm^3^ (AUC: 0.723, 95% CI: 0.674–0.768, *p* < 0.001); hematocrit > 43.3 (AUC: 0.566, 95% CI: 0.514–0.607, *p* = 0.032); NLR > 10.95 (AUC: 0.649, 95% CI: 0.598–0.698, *p* < 0.001); and NCI ≥ 6.76 (AUC: 0.749, 95% CI: 0.701–0.792, *p* < 0.001) (Figure 3). The sensitivity, specificity, PPV, NPV, and accuracy of NCI were 79.7%, 62.9%, 43.7%, 61.5%, and 80.7%, respectively (Table 6). The ability of NCI to distinguish MSAP from MAP was superior to that of NLR and hematocrit (for both, *p* < 0.01) and was similar to that of WBC (*p* = 0.13). The AUROCs of the combinations of NCI with WBC, NLR, and hematocrit (0.753, 0.750, and 0.739, respectively) were not superior to the AUROC of NCI (for all, *p* > 0.05).

Univariate analysis revealed that age, male gender, WBC, hematocrit, NLR, and NCI were significantly different between MSAP and MAP patients. In the multivariate analysis, NCI (1.119, 95% CI: 1.015–1.235, *p* = 0.023) sustained its predictive value for the prediction of the MSAP group (Table 7).

#### 3.3.3. Prognostic Accuracy of NCI and Other Laboratory Parameters to Distinguish SAP from MSAP

**Figure 4:**
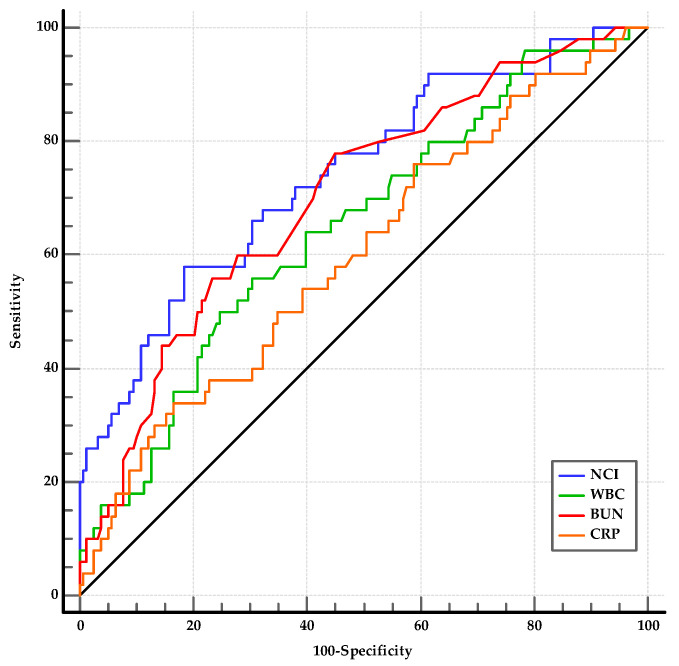
AUROCs of NCI, NLR, WBC, and hematocrit used to distinguish SAP from MSAP. BUN, blood urea nitrogen; CRP, C-reactive protein; NCI, neutrophil–creatinine index; WBC, white blood cell count.

From the laboratory parameters at admission, the WBC (*p* = 0.002), neutrophil count (*p* = 0.002), BUN (*p* < 0.001), creatinine (*p* < 0.001), CRP (*p* = 0.030), and NCI (*p* < 0.001) levels were significantly different between the SAP and MSAP groups (Table 5). Among the statistically significant laboratory parameters, WBC, BUN, CRP, and NCI were investigated using ROC analysis. The optimal cut-offs for predicting the SAP group were as follows: WBC > 14,900/mm^3^ (AUC: 0.649, 95% CI: 0.579–0.713, *p* < 0.001); BUN > 17.5 (AUC: 0.703, 95% CI: 0.621–0.785, *p* < 0.001); CRP > 81.3 (AUC: 0.602, 95% CI: 0.532–0.669, *p* = 0.029); and NCI > 15.62 (AUC: 0.742, 95% CI: 0.676–0.800, *p* < 0.001) (Figure 4). The best threshold for predicting SAP was the AUROC of NCI. Although there was no difference between the AUROCs of NCI and BUN (*p* = 0.38), NCI was more effective than WBC and CRP (for both, *p* < 0.01). The differences between WBC, BUN, and CRP were not significant (*p* > 0.05). In the comparison of the AUROCs, the combinations of WBC, BUN, and CRP with NCI (0.744, 0.755, and 0.741, respectively) did not differ from that of the AUROC of NCI (for all, *p* > 0.05) (Table 8).

As shown in Table 9, SAP was associated with age, WBC, hematocrit, BUN, CRP, NLR, and NCI in the univariate logistic regression analysis. After adjusting the prognostic factors by multivariate logistic regression, NCI (1.095, 95% CI: 1.031–1.162, *p* = 0.003) was the only independent predictor of SAP.

## 4. Discussion

The severity of AP can be predicted based on clinical, laboratory, and radiologic features and several severity scoring systems. The ideal prognostic indicators for AP should be rapid, reproducible, inexpensive, minimally invasive, and highly accurate [9]. The fact is that we need simple laboratory parameters or indices to predict high-risk patients at an earlier stage of AP. In addition, identifying mild cases will prevent overtreatment and high costs. Currently, hematocrit, BUN, creatinine, and CRP are the most reliable laboratory parameters used to evaluate severity [10]. The present study focused on the predictive values of the novel index NCI for the assessment of the clinical severity of AP, and NCI was the most predictive parameter among the studied laboratory parameters and indices that could be used to distinguish between severe cases according to BISAP and to distinguish AP subgroups according to the revised Atlanta classification. Based on the results of this study, a NCI value at admission may provide the clinician with an insight that would be helpful in predicting the disease course.

The NCI consists of two components: neutrophil count and creatinine. Neutrophils, as major immune cells associated with the active inflammation response, play an important role in the early phase of AP. They are the first recruited cells to the injury site and contribute to the local inflammatory response and necrosis in some cases. In addition to early local pancreatic events, neutrophils contribute to systemic complications and end-organ damage [11]. Activated neutrophils in AP, which have a longer lifespan and increased functional activity, are responsible for pro-inflammatory cytokine secretion, cell migration, and invasion [12]. The depletion of neutrophils leads to a significant reduction in pancreatic tissue damage [13]. We found that both the leucocyte count and the neutrophil count were capable of significantly differentiating all AP subgroups. The leucocyte count has been widely used to determine disease severity in AP as a component of Ranson’s criteria. Several studies have examined the role of neutrophil count in demonstrating the severity of AP. In a recent study by Silva-Vas et al., it was found that while the leucocyte and neutrophil counts differed between AP subgroups, the lymphocyte count did not differ [14]. Another study comparing laboratory parameters at admission for the prediction of local and systemic complication development in AP showed that leucocyte and neutrophil counts, but not lymphocyte counts, were statistically significant parameters [15]. In the mentioned study, while hemoglobin was the hemogram parameter showing the highest AUC value for the prediction of pancreatic necrosis and pseudocyst development, the neutrophil count had the highest AUC value for the prediction of systemic complications like respiratory failure and sepsis.

On the other hand, the neutrophil count has been a widely studied parameter as a component of NLR in cancers and systemic inflammatory conditions, such as acute pancreatitis. A growing number of studies have shown that NLR is a useful tool for the assessment of AP severity. Junare et al. demonstrated that NLR is the most predictive parameter among hemogram parameters and indices for ICU admission, organ failure, interventions, and mortality [16]. Another study showed that BISAP, NLR, and total calcium were independent factors for the prediction of SAP among the hemogram-derived indices, BUN, creatinine, and calcium [17]. In a meta-analysis assessing NLR for the prediction of SAP within 24 h of admission, the sensitivity and specificity values were found to be 79% and 71%, respectively, with a combined AUROC of 0.82 [18]. Our results showed that although NLR is useful in distinguishing MAP from MSAP and SAP, in multivariate analysis, NCI was the only independent predictor. Moreover, the AUROCs of the NCI were superior to the AUROCs of NLR.

The other component of NCI is creatinine. An increase in creatinine is a sign of kidney injury resulting from hypoxemia, hyperamylasemia causing impairment of renal microcirculation, a decrease in renal perfusion pressure due to abdominal compartment syndrome, intra-abdominal hypertension, hypovolemia, or systemic hemodynamic alterations, and the release of several cytokines [19,20]. Acute kidney injury (AKI) is a feared complication of SAP, carrying a poor prognosis with a high mortality rate [21]. Until recently, BUN was mainly used as an indicator of renal impairment in the assessment of AP severity. BUN is a component of BISAP, and a change in the BUN level on the second day is a criterion of Ranson’s criteria. Dai et al. showed that the BUN level at admission was the only parameter that was useful for predicting 30-day all-cause mortality [20]. Another study comparing Ranson’s score, BISAP, and several laboratory parameters at admission and 48 h after admission demonstrated that while CRP was the only predictor of SAP at admission, BUN at 48 h was the best predictor of SAP [22]. In the mentioned study, BUN at 48 h and BISAP were the best predictors of mortality, and creatinine at 48 h was the best predictor for ICU admission. Our results showed that although BUN is a useful tool for distinguishing SAP from MSAP, it was not capable of distinguishing between MAP and MSAP. BUN is affected by intravascular volume status like hemoconcentration at the time of admission or hemodilution resulting from fluid replacement therapy, and creatinine is more specific compared to BUN for the establishment of renal impairment.

Likewise, hematocrit was another laboratory parameter related to the severity of AP. The hematocrit value at the time of admission is influenced by the baseline hematocrit value, leakage into the extravascular spaces related to systemic inflammation, impaired fluid intake, and vomiting. The reversal of hemoconcentration with appropriate fluid replacement treatment indicates a favorable prognosis, with resolution of systemic inflammation. We found that hematocrit at admission was only capable of distinguishing mild cases from severe cases but not SAP from MSAP. These results may be explained by the fact that the hematocrit value at the first assessment of hospitalization may not be sufficient to distinguish between these two conditions, as the differentiation between SAP and MSAP is based on persistent organ failure associated with systemic inflammation beyond 48 h.

Advanced age is a well-known risk factor for the severity of AP. Multiorgan failure, SAP, and death are more prevalent among elderly patients compared to younger ones [23,24]. Age is one of the components of several severity indices, such as Ranson’s criteria, the Modified Glasgow Prognostic Score, and BISAP [25]. We demonstrated that disease severity was correlated with advanced age for BISAP and the subgroups of the revised Atlanta classification.

Gallstones and gallstone-related disorders are more prevalent in women. As 73% of our study population had biliary etiology, female subjects made up 60% of this population, as expected. On the other hand, we found that the disease had a milder course in females compared to males. In a large nationwide study from the USA involving 553,480 patients with AP, poor outcomes such as shock, sepsis, AKI, ICU admission, pancreatic drainage, and mortality were lower in women compared to men [26]. Another nationwide study of patients with acute biliary pancreatitis in Taiwan showed that men had a higher mortality risk, both among patients in the SAP group and in the overall population [27]. The results of these studies are consistent with our results.

In this study, it was demonstrated that NCI was capable of determining severe cases according to BISAP and was the only marker to distinguish MAP from MSAP and SAP from MSAP at admission. Although the results of NCI in predicting the severity of AP are promising, some points should be kept in mind when interpreting NCI results in clinical practice. One of them is a patient who has neutropenia. Neutropenia may develop as a systemic inflammatory response that may occur during the course of AP, or it may be detected as a result of the occurrence of AP in patients who are neutropenic at baseline due to bone marrow-related diseases or hypersplenism. False negative results for NCI may be observed in these patient groups. The other group of patients was those with chronic kidney disease with high basal creatinine values. False positive results may also be obtained in these patients.

We evaluated only NCI values at admission in this study, and serial NCI measurements were not assessed. Thus, the role of serial NCI measurements in predicting disease course and severity cannot be extrapolated based on these study results. Moreover, we could not assess the NCI in predicting long-term outcomes after hospital discharge. Since most of our patients had biliary etiology, our results cannot be generalized to all AP patients. Further prospective multicenter studies are needed to evaluate NCI’s capacity to predict disease course and severity.

The strengths of this study are that several laboratory parameters and indices were all examined together, and a new index was examined to distinguish between AP severity subgroups. Our study has several limitations. It is a retrospective and monocentric study; thus, further prospective analyses with a larger population are needed to confirm the value and to accurately measure the sensitivity and specificity. Due to the retrospective nature of the study, the patients’ data were obtained from hospital records. Long-term follow-up data and mortality data were not included in this study. We assessed the parameters at hospital admission; however, several factors related to hospital stays may influence the prognosis of AP, such as nosocomial infections.

## 5. Conclusions

In the present study, we investigated the clinical and laboratory parameters of AP patients at admission to assess disease severity in order to determine the need for treatment aggressiveness and ICU admission. Logistic regression analysis and the ROC curve proved that NCI had a high clinical predictive value. NCI was the only predictive parameter among the laboratory tests and indices that distinguished between MAP, MSAP, and SAP. This new index, which was calculated by multiplying the neutrophil count with serum creatinine, may give clues about prognosis in the early stage of AP.

## Figures and Tables

**Table 1:** Demographic and clinical characteristics of the patients.

Variable		BISAP			Revised Atlanta Classification					
	Total (*n* = 421)	Mild (*n* = 351)	Severe (*n* = 70)	*p*	MAP (*n* = 213)	MSAP (*n* = 158)	SAP (*n* = 50)	*p* _MAP-MSAP_	*p* _MAP-SAP_	*p* _MSAP-SAP_
Age (years)	62 ± 18	61 ± 17	73.5 ± 12	<0.001	60 ± 18	64 ± 19	70 ± 12	0.034	<0.001	0.007
Gender, female *n* (%)	251 (60)	219 (62)	32 (46)	0.009	144 (68)	79 (50)	28 (56)	0.001	0.121	0.459
Etiology				0.305				0.816	0.371	0.355
Biliary, *n* (%)	310 (73.6)	255 (72.6)	55 (78.6)		156 (73.2)	114 (72.2)	40 (80.0)			
Non-biliary, *n* (%)	50 (11.9)	44 (12.5)	6 (8.5)		27 (12.7)	18 (11.4)	5 (10.0)			
Alcohol	6 (1.4)	5 (1.4)	1 (1.4)		2 (0.9)	3 (1.9)	1 (2.0)			
Hypertriglyceridemia	6 (1.4)	6 (1.7)	0 (0)		3 (1.4)	3 (1.9)	0 (0.0)			
Hypercalcemia	4 (1.0)	2 (0.6)	2 (2.9)		2 (0.9)	0 (0.0)	2 (4.0)			
Drugs	13 (3.1)	11 (3.1)	2 (2.9)		8 (3.8)	4 (2.5)	1 (2.0)			
Infections	10 (2.4)	10 (2.8)	0 (0)		7 (3.3)	3 (1.9)	0 (0.0)			
IPMN	5 (1.2)	5 (1.4)	0 (0)		2 (0.9)	3 (1.9)	0 (0.0)			
Post-ERCP	3 (0.7)	2 (0.6)	1 (1.4)		1 (0.5)	1 (0.6)	1 (2.0)			
Pancreas divisum	3 (0.7)	3 (0.9)	0 (0)		2 (0.9)	1 (0.6)	0 (0.0)			
Idiopathic	61 (14.5)	52 (14.8)	9 (12.9)		30 (14.1)	26 (16.4)	5 (10.0)			
Ranson, median (IQR)	2 (3)	2 (2)	5 (2)	<0.001	2 (2)	3 (2)	6 (3)	<0.001	<0.001	<0.001
Death, *n* (%)	18 (4.3)	2 (0.6)	16 (23)	<0.001	0 (0)	2 (1.3)	16 (32)	0.181	<0.001	<0.001

ERCP, endoscopic retrograde cholangiopancreatography; IPMN, intraductal papillary mucinous neoplasia.

**Table 2:** Comparisons of laboratory parameters of patients with acute pancreatitis according to BISAP score.

Variable	Total (*n* = 421)	Mild AP (*n* = 351)	Severe AP (*n* = 70)	*p*
WBC (×10^3^/μL)	11.72 ± 0.25	11.41 ± 4.25	16.84 ± 6.45	<0.001
Neutrophil (×10^3^/μL)	9.27 ± 0.24	7.55 ± 0.27	11.11 ± 0.35	<0.001
Lymphocyte (×10^3^/μL)	1.13 ± 0.04	1.17 ± 0.05	1.07 ± 0.07	0.238
Hemoglobin (g/dL)	13.4 ± 1.9	13.3 ± 1.8	14.1 ± 2.5	0.017
Hematocrit (%)	40.1 ± 5.6	39.7 ± 5.1	42.4 ± 6.9	0.002
Platelet (×10^3^/μL)	254 ± 82	254 ± 79	255 ± 95	0.487
ALT (U/L)	186 ± 10.1	186 ± 12.2	173 ± 16.1	0.737
AST (U/L)	188 ± 12.8	189 ± 15.3	185 ± 20.5	0.724
Calcium (mg/dL)	9.2 ± 0.7	9.2 ± 0.6	9.1 ± 1.0	0.154
BUN (mg/dL)	17 ± 0.76	15 ± 0.78	18 ± 1.31	<0.001
Creatinine (mg/dL)	0.87 ± 0.02	0.80 ± 0.02	0.96 ± 0.04	<0.001
Amylase (U/L)	1422 ± 55	1334 ± 85	1490 ± 69	0.009
Lipase (U/L)	1558 ±117	1449 ± 156	1711 ± 176	0.691
CRP (mg/L)	13.8 ± 3.0	10.8 ± 3.4	16.1 ± 5.0	0.001
NLR	8.33 ± 0.62	6.30 ± 0.70	12.1 ± 0.99	<0.001
PLR	220 ± 11.0	199 ± 14.6	244 ± 16.5	0.287
NCI	8.03 ± 0.53	5.99 ± 0.31	10.89 ± 0.95	<0.001

ALT, alanine aminotransferase; AST, aspartate aminotransferase; BUN, blood urea nitrogen; CRP, C-reactive protein; NCI, neutrophil–creatinine index; NLR, neutrophil to lymphocyte ratio; PLR, platelet to lymphocyte ratio; WBC, white blood cell count.

**Table 3:** ROC curve analysis results for the prediction of severe AP according to BISAP.

Variable	Cut-Off	AUROC	95% CI	Sensitivity (%)	Specificity (%)	PPV (%)	NPV (%)	Accuracy (%)	*p*
WBC	11.88	0.788	0.746–0.826	85.7	60.4	30.1	95.5	64.6	<0.001
BUN	17.8	0.785	0.742–0.823	84.3	60.4	29.8	95.1	64.4	<0.001
Hct	43.3	0.623	0.576–0.669	48.6	79.2	31.8	88.5	74.1	0.004
CRP	61.45	0.629	0.581–0.676	40.0	81.5	30.1	87.2	74.6	<0.001
NLR	8.42	0.689	0.642–0.732	77.1	55.8	25.7	92.4	59.1	<0.001
NCI	11.27	0.862	0.826–0.894	78.6	79.8	43.7	94.9	79.6	<0.001
NCI + WBC		0.862	0.825–0.893	80.0	77.8	41.8	95.1	78.2	<0.001
NCI + NLR		0.862	0.826–0.894	80.0	79.2	43.4	95.2	79.3	<0.001
NCI + Hct		0.862	0.826–0.894	84.3	75.8	41.0	96.0	77.2	<0.001
NCI + BUN		0.877	0.841–0.906	87.4	74.1	40.1	96.7	76.3	<0.001
NCI + CRP		0.862	0.826–0.894	78.6	80.3	44.4	95.0	80.1	<0.001

AUROC, area under the receiver operating characteristics curve; BUN, blood urea nitrogen; CI, confidence interval; CRP, C-reactive protein; Hct, hematocrit; NCI, neutrophil–creatinine index; NLR, neutrophil to lymphocyte ratio; NPV, negative predictive value; PPV, positive predictive value; WBC, white blood cell count.

**Table 4:** Logistic regression analyses for variables associated with severe disease according to BISAP.

	*Univariate*			*Multivariate*		
Variable	OR	95% CI	*p*	OR	95% CI	*p*
Age	1.012	1.001–1.024	0.003	1.046	1.018–1.074	0.001
Gender, female	0.508	0.303–0.852	0.01	0.812	0.401–1.646	0.564
WBC	1.232	1.159–1.309	<0.001	1. 141	1.026–1.269	0.015
Hct	1.095	1.043–1.149	0.033	1.081	1.015–1.152	0.015
BUN	1.058	1.038–1.078	<0.001	1.040	1.014–1.066	0.002
CRP	1.006	1.003–1.010	<0.001	0.994	0.970–1.019	0.282
NLR	1.178	1.126–1.232	<0.001	1.004	0.980–1.029	0.662
NCI	1.041	1.131–1.257	<0.001	1.076	1.014–1.142	0.015

BUN, blood urea nitrogen; CRP, C-reactive protein; Hct, hematocrit; NCI, neutrophil–creatinine index; NLR, neutrophil to lymphocyte ratio; WBC, white blood cell count.

**Table 5:** Laboratory parameters of patients with AP at hospital admission.

Variable	MAP (*n* = 213)	MSAP (*n* = 158)	SAP (*n* = 50)	*p* _MAP-MSAP_	*p* _MAP-SAP_	*p* _MSAP-SAP_
WBC (×10^3^/μL)	9.58 ± 0.28	12.85 ± 0.33	15.31 ± 0.10	<0.001	<0.001	0.002
Neutrophil (×10^3^/μL)	7.55 ± 0.27	10.69 ± 0.33	12.72 ± 0.95	<0.001	<0.001	0.002
Lymphocyte (×10^3^/μL)	1.17 ± 0.05	1.10 ± 0.09	1.00 ± 0.12	0.291	0.178	0.595
Hemoglobin (g/dL)	13.2 ± 1.7	13.7 ± 2.0	13.9 ± 2.7	0.006	0.070	0.567
Hematocrit (%)	39.2 ± 4.7	40.8 ± 5.7	41.9 ± 7.2	0.005	0.015	0.245
Platelet (×10^3^/μL)	248 ± 79	259 ± 78	259 ±104	0.184	0.483	0.997
ALT (U/L)	186 ± 12.2	154 ± 19.9	215 ± 23.3	0.521	0.815	0.542
AST (U/L)	189 ± 15.3	171 ± 25.1	190 ± 31.3	0.658	0.642	0.448
Calcium (mg/dL)	9.2 ± 0.6	9.1 ± 0.6	9.2 ± 1.1	0.881	0.956	0.988
BUN (mg/dL)	15 ± 0.78	17 ± 1.04	26 ± 4.05	0.144	<0.001	<0.001
Creatinine (mg/dL)	0.80 ± 0.02	0.91 ± 0.03	1.21 ± 0.12	<0.001	<0.001	<0.001
Amylase (U/L)	1334 ± 85	1473 ± 73	1702 ± 167	0.199	0.022	0.110
Lipase (U/L)	1449 ± 156	1718 ± 188	1568 ± 428	0.094	0.616	0.698
CRP (mg/L)	10.8 ± 3.4	15.4 ± 5.0	25.5 ± 13.4	0.102	0.001	0.030
NLR	6.30 ± 0.70	11.66 ± 0.91	13.88 ± 2.92	<0.001	<0.001	0.102
PLR	199 ± 14.6	244 ± 17.1	239 ± 42.7	0.051	0.178	0.989
NCI	5.99 ± 0.31	9.85 ± 0.51	17.19 ± 3.27	<0.001	<0.001	<0.001

ALT, alanine aminotransferase; AST, aspartate aminotransferase; BUN, blood urea nitrogen; CRP, C-reactive protein; NCI, neutrophil–creatinine index; NLR, neutrophil to lymphocyte ratio; PLR, platelet to lymphocyte ratio; WBC, white blood cell count.

**Table 6:** ROC curve analysis results for the prediction of MSAP.

Variable	Cut-Off	AUROC	95% CI	Sensitivity (%)	Specificity (%)	PPV (%)	NPV (%)	Accuracy (%)	*p*
WBC	10.84	0.723	0.674–0.768	73.4	65.3	61.0	76.8	68.7	<0.001
NLR	10.95	0.649	0.598–0.698	55.7	79.9	69.4	68.8	69.0	<0.001
Hct	43.3	0.566	0.514–0.607	32.9	83.6	59.8	62.7	62.0	0.032
NCI	6.76	0.749	0.701–0.792	79.7	62.9	61.5	80.7	70.1	<0.001
NCI + WBC		0.753	0.706–0.796	82.3	60.1	60.5	82.1	69.5	<0.001
NCI + NLR		0.750	0.703–0.793	81.0	61.5	60.9	81.4	69.8	<0.001
NCI + Hct		0.739	0.691–0.783	71.5	67.6	62.1	76.2	69.3	<0.001

AUROC, area under the receiver operating characteristics curve; CI, confidence interval; Hct, hematocrit; NCI, neutrophil–creatinine index; NLR, neutrophil to lymphocyte ratio; NPV, negative predictive value; PPV, positive predictive value; WBC, white blood cell count.

**Table 7:** Logistic regression analyses for variables associated with MSAP.

	*Univariate*			*Multivariate*		
Variable	OR	95% CI	*p*	OR	95% CI	*p*
Age	1012	1.001–1.024	0.003	1.003	0.988–1.018	0.660
Gender, female	0.479	0.314–0.479	0.001	0.730	0.427–1.248	0.250
WBC	1.202	1.135–1.273	<0.001	1.085	0.980–1.200	0.116
Hct	1.059	1.016–1.103	0.005	1.029	0.982–1.079	0.277
BUN	1.011	0.994–1.028	0.182	-	-	-
CRP	1.002	0.998–1.006	0.164	-	-	-
NLR	1.043	1.021–1.065	<0.001	1.004	0.980–1.029	0.660
NCI	1.193	1.131–1.257	<0.001	1.119	1.015–1.235	0.023

BUN, blood urea nitrogen; CRP, C-reactive protein; Hct, hematocrit; NCI, neutrophil–creatinine index; NLR, neutrophil to lymphocyte ratio; WBC, white blood cell count.

**Table 8:** ROC curve analysis results for the prediction of SAP.

Variable	Cut-Off	AUROC	95% CI	Sensitivity (%)	Specificity (%)	PPV (%)	NPV (%)	Accuracy (%)	*p*
WBC	14.90	0.649	0.579–0.713	56.0	69.6	36.8	83.3	66.3	<0.001
BUN	17.5	0.703	0.621–0.785	78.0	54.4	35.1	88.7	60.1	<0.001
CRP	81.3	0.602	0.532–0.669	34.0	82.3	37.8	79.7	70.7	0.029
NCI	15.62	0.742	0.676–0.800	58.0	81.0	49.1	85.9	75.5	<0.001
NCI + WBC		0.744	0.679–0.802	54.0	84.8	52.9	83.3	77.4	<0.001
NCI + BUN		0.755	0.691–0.812	66.0	75.3	45.8	87.5	73.1	<0.001
NCI + CRP		0.741	0.676–0.799	58.0	81.6	50.0	86.0	75.9	<0.001

AUROC, area under the receiver operating characteristics curve; BUN, blood urea nitrogen; CI, confidence interval; CRP, C-reactive protein; NCI, neutrophil–creatinine index; NPV, negative predictive value; PPV, positive predictive value; WBC, white blood cell count.

**Table 9:** Logistic regression analyses for variables associated with SAP.

	*Univariate*			*Multivariate*		
Variable	OR	95% CI	*p*	OR	95% CI	*p*
Age	1.022	1.001–1.043	0.035	1.008	0.982–1.034	0.545
Gender, female	1.273	0.671–2.413	0.459	-	-	-
WBC	1.118	1.048–1.193	0.001	0.975	0.869–1.093	0.663
Hct	1.031	0.978–1.087	0.030	1.006	0.946–1.070	0.850
BUN	1.037	1.016–1.058	<0.001	1.015	0.989–1.040	0.258
CRP	1.004	1.000–1.008	0.020	0.999	0.994–1.005	0.786
NLR	1.022	1.001–1.044	0.037	1.001	0.975–1.028	0.959
NCI	1.099	1.057–1.142	<0.001	1.095	1.031–1.162	0.003

BUN, blood urea nitrogen; CRP, C-reactive protein; Hct, hematocrit; NCI, neutrophil–creatinine index; NLR, neutrophil to lymphocyte ratio; WBC, white blood cell count.

## Data Availability

The data presented in this study are available on request from the corresponding author.
